# Global trends in lifespan inequality: 1950-2015

**DOI:** 10.1371/journal.pone.0215742

**Published:** 2019-05-02

**Authors:** Iñaki Permanyer, Nathalie Scholl

**Affiliations:** Centre d’Estudis Demogràfics (a member of the CERCA Programme / Generalitat de Catalunya), Cerdanyola del Vallès, Barcelona, Spain; University of North Texas Health Science Center, UNITED STATES

## Abstract

Using data from the UN World Population Prospects, we document global trends in lifespan inequality from 1950 until 2015. Our findings indicate that (i) there has been a sustained decline in overall lifespan inequality, (ii) adult lifespan variability has also declined, but some plateaus and trend reversals have been identified, (iii) lifespan inequality among the elderly has *increased* virtually everywhere, and (iv) most of the world variability in age-at-death can be attributed to *within-*country variability. Such changes have occurred against a backdrop of generalized longevity increases. Our analyses suggest that the world is facing a new challenge: the emergence of diverging trends in longevity and age-at-death inequality among the elderly around the globe—particularly in high-income areas. As larger fractions of the world population survive to more advanced ages, it will be necessary for national and international health planners to recognize the growing heterogeneity that characterizes older populations.

## Introduction

Living a long and healthy life is among the most highly valued and universal human goals, so the unparalleled longevity gains recorded all over the world during the last few decades are cause for celebration. While a huge body of scholarship has shed considerable light on the ‘efficiency part’ of the process (i.e., the global, regional and national trajectories in life expectancy over time are very well documented [1–2]), much less is known about the ‘equality part’. Since mortality can arguably be considered the ultimate measure of health, lifespan inequalities should be seen as the most fundamental manifestations of health disparities. Indeed, the existence of very unequal length of life distributions might go beyond purely natural causes and could be indicative of an unfair state of affairs in which some population groups might be disadvantaged or discriminated against. For this reason, the study of lifespan variability has attracted a great deal of attention from demographers and other social scientists, particularly during the last decade or so [3–15].

Studies on lifespan disparities usually focus their attention on differences occurring either *between* or *within* countries. The former approach typically compares the average health performance among a cross-section of countries (most often by comparing the corresponding life expectancies) and aims at understanding why population health is better in some countries than in others [16–18]. In contrast, the latter approach explores the lifespan differences that might exist among the individuals within a given country. Surprisingly, the study of *global* lifespan inequality (henceforth referred to as GLI)—that is, the study of variations in individuals’ lifespan both within *and* between all world countries (henceforth WLI and BLI, respectively)—is largely underdeveloped; so far, GLI has only been analyzed in a few studies using either one or two cross-sections in time ([4, 19]). Despite the importance of GLI, our understanding of how the different types of inequalities are articulated into a coherent whole and how their relationship evolves as the demographic transition unfolds is still in its infancy—an issue we aim to address in this paper. For the first time, we document the joint evolution in within-country, between-country and global lifespan inequality during the period spanning from 1950 to 2015, and we investigate in detail the relationship between these trends and the advances in longevity that are sweeping the world.

There are many reasons to be interested in the study of *global* trends in length of life inequality. First, from a practical perspective, we now have the ability to do so. Not long ago, the set of life tables needed to conduct comparative analyses across and within world countries for long time periods of time was very difficult for researchers to access. Second, from an ethical perspective, if all human beings are entitled to equal rights, egalitarian concerns should apply equally at the national *and* global levels (e.g., should we allow an individual’s lifespan prospects to be determined by his or her country of birth?). Third, the study of global inequalities allows us to see how the world has changed—often in fundamental ways—and to study the hotly debated consequences of economic globalization or other global phenomena affecting the living conditions of all human beings. Finally, exploring how age-at-death differentials jointly evolve within and between countries can shed considerable light on our understanding of human mortality processes and improve the quality of national and international public health policies.

An analysis of global lifespan inequality must necessarily take into consideration the unprecedented demographic transformations undergone by the world and its regions since the 1950s. On the one hand, the unfolding of demographic and epidemiological transitions during more than six decades has dramatically changed countries’ population structures across the globe. While the prevalence of infant mortality was particularly high among most world countries in the mid-20^th^ century, currently, childhood and reproductive-age mortality have shrunk considerably, thus moving age-at-death distributions to more advanced ages. On the other hand, country-specific life expectancies and the corresponding population shares (which, in turn, are affected by differential population growth trajectories) strongly influence global trends in lifespan inequality. To gauge the specific effect that such structural changes have had on lifespan variability across and within countries, we incorporate the following analytical strategies. First, we study lifespan variability, not only across the entire age range but also across adult *and* more advanced age ranges. For convenience, the last two ranges comprise the ages above 15 and above 65, respectively (similar age thresholds have been used in many conceptually related papers [4–5, 19]); the substantive findings of the paper remain unaffected by the choice of alternative thresholds). As suggested in previous studies, there are good reasons to separate childhood, adult and elderly mortality [3–5, 19]. Second, we resort to well-known and newly developed inequality decomposition techniques that allow going beyond purely descriptive results to analyze which factors are the most important drivers of lifespan dispersion and its evolution over time. Inter alia, we run several counterfactual analyses to identify the influence that countries’ relative population size, longevity and within-country lifespan inequality have had on the dynamics of global lifespan inequality.

The empirical analysis relies on the latest version of the United Nations’ World Population Prospects (WPP) for the period from 1950 to 2015. The widespread geographic coverage of the database (195 countries) allows global, regional and country-level analyses to be performed over time. Based on this data source, this paper aims to (i) document levels and trends in longevity and lifespan inequality for overall adult and elderly mortality in the world and its regions; (ii) decompose global lifespan inequality in its within- and between-country components and assess what the corresponding contributions are; and (iii) examine the main potential sources of lifespan inequality within and across countries.

### Longevity and lifespan variation

Classical health transition theories suggest that longevity increases go in tandem with a transformation of the mortality distribution characterized by a concentration of deaths around the modal age at death, with both the mean and modal age at death increasing [20–21]. In this line, the so-called ‘mortality compression’ or ‘rectangularization hypothesis’ popularized by Fries [22] postulates that as the epidemiological transition unfolds, the human survival curves gradually adopt a rectangular shape as life expectancy at birth increases and approaches an upper limit of the human lifespan. In the limit, the survival curve would become fully rectangular, and all deaths would occur at the same age. While Dong et al [23] suggest that the maximum lifespan of humans is fixed and is unlikely to increase over time, most empirical evidence has shown no evidence of an upper bound to life expectancy, which continues to increase unabated [2, 24].

The progressive rectangularization of the survival curve has been observed in several high-income countries [25–26]. In the majority of cases, increasing longevity is associated with low lifespan disparities when one considers the entire range of ages at death [6]. However, several studies have noted that by restricting our attention to selected age ranges, the relationship between longevity and lifespan variability weakens and even reverses. For the US, [27] found that while the relationship between life expectancy and lifespan inequality across the entire age range was negative, the relationship turned positive when the age at death distribution was bottom-truncated at the age of 60. Nusselder and Mackenbach [28], Robine, [29] and Engelman, Canudas-Romo and Agree [5] found similar patterns for other highly industrialized countries. In the same context, Edwards and Tuljapurkar [3] found stagnating—rather than the expected declining—trends in lifespan variability when bottom truncating the lifespan distribution at the age of 10. While the selection of specific age ranges in the study of mortality compression has been criticized on grounds of arbitrariness ([29], page 187), several authors suggest that studying variability measures *conditional* upon survival to a certain age (i.e., exploring the so-called ‘conditional’ age-at-death distributions) is a promising avenue of research that can reveal unexpected patterns in adult mortality that are otherwise concealed by unconditional measures [5, 21]. As longevity increases and larger fractions of the population survive to more advanced ages, it becomes important to go beyond the analysis of mortality across the entire age range and focus our attention on some of its subsets. Rather than sticking to a particular age range, in this paper, we document global trends in overall, adult and elderly lifespan variability.

Current evidence on *global* length of life inequality and its between- and within-country subcomponents is still incomplete. In general, unconditional length of life inequality within countries has tended to decrease as longevity increases [4, 6]. However, Engelman, Canudas-Romo and Agree [5] report increases in lifespan variability among the elderly within a group of high-income countries. Regarding between-country variation, some cross-national studies have reported worldwide convergence in life expectancy levels between the 1950s and the late 1980s [18, 30]. Unfortunately, the spread of HIV/AIDS in Africa and the collapse of Communism contributed to reversing this favorable trend. Finally, evidence on global trends in lifespan inequality is particularly scarce. Using life tables from 180 countries, [19] shows that world inequality in length of life diminished between 1970 and 2000. In line with the previous two studies, his findings suggest that between-country inequality increased between the two points in time—a matter of concern for public health planners. One of the main aims of this paper is to expand the scope of previous studies by providing a detailed account of the global trends in lifespan inequality for the world and its regions during the last 65 years. In our analysis, we will explore both unconditional and conditional age-at-death distributions.

### Data

The main data source employed in this paper is the UN World Population Prospects’ (WPP) abridged life tables, which record the number of deaths for age groups in 5-year intervals (with separate data for infants (age group 0–1) as well as an open-ended 100+ interval). We aggregate our estimates for both sexes, but data are also available separately for females and males (see section 4.4). The life tables information is complemented with countries’ population size (also available from UN’s WPP), which is needed to calculate the between-country component of global lifespan inequality. We use life tables from 195 countries over thirteen 5-year time periods (from 1950–55 until 2010–15), yielding a total of 2535 country-period observations. To facilitate the presentation of results, we aggregate the data at different levels employing the standard United Nations’ regional classification of countries (see S1 File). Due to the marked impact of the HIV-AIDS epidemic on length-of-life distributions, we create a separate category for Sub-Saharan African countries that have had an HIV prevalence of more than 3%.

While there are excellent data on mortality by age group for high-income countries, data are generally sparser and less reliable for developing countries. Nevertheless, the UN population division has assembled a broad data set of country life tables and provides a detailed account of the data sources used in the construction of each country’s set of mortality estimates (see https://esa.un.org/unpd/wpp/DataSources/). Although the use of model life tables is unavoidable for constructing complete data series for all developing countries, all missing country-year combinations are estimated via indirect methods based on real data. Therefore, while the accuracy of individual inequality estimates might not be perfect for every country in every year, we have compelling reasons to believe that the overall picture that emerges from them is a faithful portrait of reality. As indicated in this paper, our empirical findings square well with those from other renowned studies, and the estimates we obtain from the UN WPP are highly correlated with the estimates derived from other reputable data sources, such as the Human Mortality Database (HMD)—a fact that can be attributed to the similarity of methods that both sources employ to generate their estimates.

## Methods

### Measuring lifespan inequality

Currently, there is an unsettled debate on whether lifespan inequality should be measured using absolute or relative measures (sometimes also referred to as ‘additive’ and ‘proportional’ measures, respectively). While there is a long tradition in using relative inequality measures (partly driven by their widespread use among economists because of their ability to compare income distributions expressed in different currencies), there is no theoretical reason why one should disregard the use of absolute measures when exploring differences in length of life. The choice between absolute and relative measures can be problematic when assessing trends because the corresponding results do not necessarily coincide—an issue that is partly attributable to the explicit dependence of relative measures to the values of the mean, which tend to change over time. Very often, relative measures might show declines because the mean has increased, while absolute measures might remain unaffected—a technical point that should be taken into consideration when interpreting the results. Since the choice between both kinds of measures is purely normative [31] and no clear consensus seems to be in place, in this paper we use both absolute and relative inequality measures.

In the last few years, several measures have been proposed to measure lifespan variability (see Wrycza, Missov and Baudisch [32] for an excellent review of the most widely used measures). We selected specific inequality indices based on their popularity and decomposability properties, which, as we show below, are very useful for the purposes of this paper. The first measure we consider is the Theil index, which we denote as *T*_*a*_ (where *a* is the youngest age interval taken from the life table; the formula of the Theil index is shown in S1 File). When *a* = 0, we include the entire lifespan distribution, and when *a* = 15, we disregard mortality under 15 and focus on adult mortality only. Since both approaches have been used in the literature (see Smits and Monden [4] and Edwards [19]), we calculate inequality statistics for both the unconditional and the conditional distributions. In addition, we also investigate lifespan inequality trends when *a* = 65, that is, length of life inequality among the population beyond the standard retirement age—an analysis that, so far, has only been conducted in a reduced group of high-income countries (see Engelman, Canudas-Romo and Agree [5]).

Another one of the inequality indices we will consider in the paper is the variance, which we denote as *V*_*a*_ (the formula of the variance is shown in S1 File). Unlike the Theil index, the variance is an absolute inequality measure. Again, we will report the values of this inequality measure for *a* = 0, *a* = 15 and *a* = 65. As a robustness check, in S1 File, we complement our analysis showing the results arising from other well-known inequality measures, such as the Gini index or the coefficient of variation.

Like all inequality measures, *T*_*a*_ and *V*_*a*_ measure the degree of dispersion in a given distribution. In the context analyzed in this paper, both measures decrease (resp. increase) when the individuals in a given society tend to die at increasingly similar (resp. dissimilar) ages.

### Inequality decompositions

The reason why we have chosen the Theil index and the variance is that they are amenable to interesting decompositions that can shed some light on the factors behind lifespan variability dynamics. The Theil index and the variance are known for their additive decomposability property. This means that global lifespan inequality (i.e., variations in age at death around the whole world) can be broken down into two clearly interpretable components: the inequality observed *within* countries and the component capturing the differences in average attainment *between* countries. More formally, additively decomposable inequality measures can be written as

$$I\;=\;I_{B}+I_{W}$$ (1)

In the last equation, *I*_*B*_ represents the inequality that would be observed in a hypothetical distribution (sometimes referred to as a ‘smoothed distribution’) where the age at death of each individual corresponds to the average age at death in the corresponding country (i.e., eliminating within-country variations). The second term is a weighted average of lifespan inequality within countries. The decomposition formula (1) can be applied irrespective of the choice of the age range (i.e., both for conditional and unconditional lifespan distributions—see S1 File for a full development of this equation).

### Lifespan variation counterfactuals

As shown in S1 File, global lifespan inequality is a function of three factors: (i) population shares (*s*), (ii) longevity (*μ*), and (iii) lifespan variability (*I*) in the different world countries. To simplify notation and explicitly indicate the dependency of lifespan inequality on these three factors, we will schematically rewrite equation (Eq 1) as

$$I_{t}\;=\;f(\left\{s_{t}\},\{\mu_{t}\},\{I_{t}\right\})$$ (2)

where the bold letters indicate the countrywide vectors of population shares, longevity and within-country inequality, respectively. The subscript ‘*t*’ now refers to the time period and *f* is a function (in S1 File, we show the specific functional form that (Eq 2) adopts when applied to the cases of the Theil index and the variance). Given the important transformations undergone by these three components around the world during recent decades, it is interesting to gauge their relative importance in assessing changes in overall lifespan inequality over time. To address this issue, we use a set of counterfactual analyses. We ask what would have happened to total lifespan inequality in time period ‘2’ if we held constant one of the three quantities that appear in the inequality index at its earlier (time period ‘1’) value and allowed the other two to take their later (time period ‘2’) value. In this way, we generate a counterfactual level of lifespan inequality in time period ‘2’, and by comparing this with observed inequality in time period ‘2’, we can assess the impact of change in the quantity we kept fixed at the time ‘1’ level on inequality. In this way, we generate the following counterfactual inequalities:

$$C_{1}\;=\;f(\left\{s_{1}\},\{\mu_{2}\},\{I_{2}\right\})$$ (3)

$$C_{2}\;=\;f(\left\{s_{2}\},\{\mu_{1}\},\{I_{2}\right\})$$ (4)

$$C_{3}\;=\;f(\left\{s_{2}\},\{\mu_{2}\},\{I_{1}\right\})$$ (5)

Hence, *C*_1_ indicates the level of lifespan inequality we would observe in time ‘2’ if the population shares of each country remained at their time ‘1’ levels (i.e., if there were zero population growth). The second counterfactual measures the level of inequality we would observe in time ‘2’ in case the longevity in each country had not changed over time. Lastly, *C*_3_ measures the inequality we would observe in time ‘2’ if within-country lifespan variation had remained at its time ‘1’ levels. Comparing the values of the counterfactuals *C*_1_, *C*_2,_ and *C*_3_ with the observed inequality levels (i.e., *I*_1_ = *f* ({***s***_1_}, {***μ***_1_}, {***I***_1_}) and *I*_2_ = *f* ({***s***_2_}, {***μ***_2_}, {***I***_2_}) we can estimate which of the three factors might have been more decisive in driving lifespan inequality changes over time. Clearly, the counterfactuals shown in Eqs (1) to (5) can be computed both for conditional and unconditional lifespan distributions.

## Empirical findings

### Regional trends

**Fig 1:**
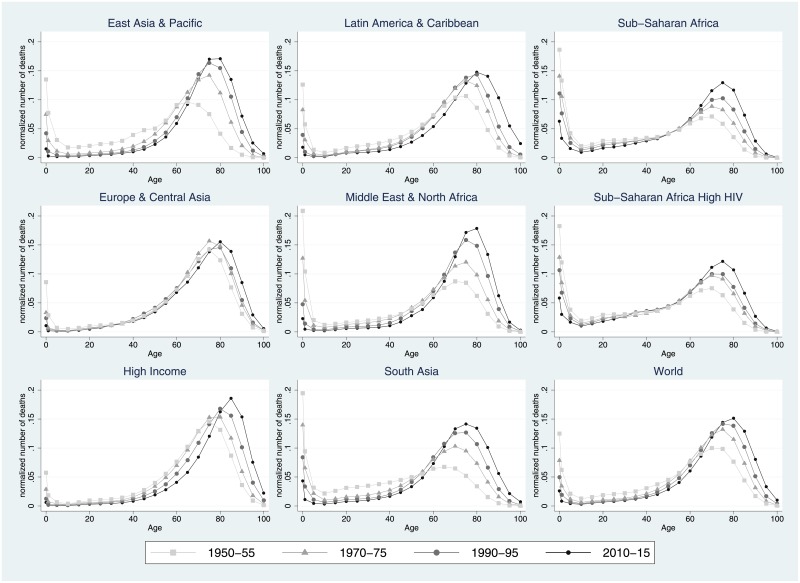
Density functions with age-at-death distributions in 1950–55, 1970–75, 1990–95, and 2010–15 in the world as a whole and in its regions. EAP = East Asia & Pacific, CA = Central Asia, HIC = High-income countries, LAC = Latin America & Caribbean, MENA = Middle East & North Africa, SA = South Asia, SSH-HIV = Sub-Saharan Africa High HIV, SSA = Other Sub-Saharan African countries. Source: Authors’ calculations based on UN World Population Prospects data.

In the different panels of Fig 1, we show the evolution of length of life distributions between 1950–55 and 2010–15 for the world as a whole and for its different regions. Two major changes occurred between the mid-20^th^ century and the present. First, all distributions have clearly shifted to the right, thus indicating a lengthening of lifespan across all of the regions and for the world as a whole. Second, the shape of the age-at-death distributions has changed dramatically during recent decades. In the 1950s, age-at-death distributions were twin-peaked, with a local/global maximum for the first age bracket and another local/global maximum at an adult age varying across regions. With the unfolding of the epidemiological and demographic transitions, infant mortality has decreased dramatically, thus gradually shifting the age at death distributions towards the right and increasingly concentrating deaths around their modal age. While these trends generally apply to all regions, we observe substantial heterogeneity across them. After World War II, the child mortality peak of the age-at-death distributions was higher than the adult mortality peak in all world regions except for the group of high-income countries, where child mortality levels were already very low in the 1950s. In the following decades, improvements in the age-at-death distributions can be observed across the board, but the pace of change has not been the same everywhere. In particular, we observe some stagnation around the 1990s for Central Asia and in the HIV-stricken countries of Sub-Saharan Africa.

In light of the aforementioned transformations, what is the extent of longevity and lifespan inequality of the age-at-death distributions shown in Fig 1? The results, presented in Table 1, show several patterns that are worth pointing out. Regarding unconditional lifespan distributions, global and regional life expectancies at birth have tended to increase monotonically all over the world (see the first group of columns in Table 1). The group of high-income countries has always had the highest longevity (regional *e*_0_ of 65 in 1950–55 up to 78.6 in 2010–15). At the other extreme, Sub-Saharan Africa is the region with the lowest life expectancy across all periods (except in 1950–55, when South Asia was the region with the lowest regional *e*_0_). In tandem with these increases in longevity, we also observe monotonic declines in unconditional lifespan inequality at all time periods and in all places (no matter which inequality measure we choose)—a finding that aligns well with conceptually related studies [6]. Given the strong relationship between life expectancy at birth and unconditional lifespan inequality, it is not surprising to find the group of high-income countries and Sub-Saharan Africa as the regions with the lowest and highest length of life inequality across the studied period.

**Table 1:** Regional indicators of longevity and lifespan inequality for unconditional and conditional age-at-death distributions.

Region	Year	Pop. (mio.)	Full lifespan			Ages 15+			Ages 65+		
			*e*_0_	Theil	Var	*μ*_15_	Theil	Var	*μ*_65_	Theil	Var
EAP	1950–55	732.7	44.6	0.298	854.0	59.4	0.049	308.4	74.6	0.0036	40.9
	1970–75	1137.1	60.6	0.141	672.4	68.6	0.027	228.5	77.1	0.0041	49.3
	1990–95	1659.3	68.8	0.077	473.4	73.0	0.021	199.7	78.9	0.0045	56.8
	2010–15	2005.0	74.2	0.038	299.3	75.8	0.018	186.2	80.6	0.0050	65.2
CA	1950–55	18.1	55.0	0.215	869.4	66.7	0.037	295.0	77.9	0.0051	62.6
	1970–75	34.4	62.4	0.149	753.7	70.9	0.030	268.5	79.5	0.0055	70.6
	1990–95	51.2	65.3	0.107	597.8	71.0	0.028	254.2	79.4	0.0053	67.8
	2010–15	63.9	70.0	0.057	388.4	72.6	0.023	217.3	79.4	0.0051	65.1
HIC	1950–55	854.0	65.0	0.105	576.3	70.6	0.027	235.3	78.4	0.0045	55.4
	1970–75	1046.1	71.1	0.049	350.9	73.2	0.022	212.8	79.6	0.0048	60.6
	1990–95	1184.9	74.3	0.036	305.1	75.5	0.022	226.4	81.5	0.0053	69.7
	2010–15	1275.7	78.6	0.026	256.7	79.2	0.019	214.8	84.1	0.0053	74.8
LAC	1950–55	168.7	52.0	0.243	900.1	64.8	0.043	323.4	77.3	0.0044	52.6
	1970–75	288.1	61.6	0.146	728.1	69.7	0.032	273.7	78.8	0.0048	60.6
	1990–95	448.5	68.8	0.078	500.3	72.5	0.029	265.5	80.3	0.0053	69.2
	2010–15	603.4	74.8	0.050	403.5	76.8	0.026	266.1	83.1	0.0061	84.4
MENA	1950–55	92.5	43.4	0.387	1058.1	63.5	0.046	327.5	76.5	0.0039	46.2
	1970–75	155.3	55.2	0.228	921.4	68.2	0.034	279.4	77.9	0.0043	52.7
	1990–95	261.2	66.7	0.097	559.7	72.1	0.025	226.6	79.0	0.0044	55.1
	2010–15	383.8	72.5	0.050	361.3	74.9	0.021	203.4	80.4	0.0046	59.5
SA	1950–55	477.0	37.2	0.411	880.3	55.4	0.065	367.7	75.0	0.0038	43.8
	1970–75	715.3	49.6	0.274	931.9	64.3	0.041	303.0	76.6	0.0044	52.0
	1990–95	1151.0	59.8	0.156	734.2	68.6	0.031	260.1	77.7	0.0047	57.2
	2010–15	1652.3	68.2	0.083	512.4	72.5	0.026	240.4	79.6	0.0054	68.4
SSH-HIV	1950–55	122.8	37.3	0.428	927.5	56.9	0.064	372.8	74.9	0.0034	39.0
	1970–75	193.5	46.2	0.301	928.7	61.4	0.053	354.2	76.1	0.0039	45.6
	1990–95	334.5	49.7	0.250	878.3	62.2	0.052	360.1	76.8	0.0041	49.2
	2010–15	558.1	58.1	0.149	708.5	65.3	0.046	348.1	78.0	0.0044	54.5
SSA	1950–55	58.6	35.8	0.453	922.5	56.4	0.065	373.7	74.8	0.0034	38.6
	1970–75	92.0	43.3	0.346	961.9	60.4	0.057	368.3	76.1	0.0039	45.3
	1990–95	161.8	49.5	0.266	926.8	63.3	0.051	359.7	77.1	0.0041	49.5
	2010–15	290.5	59.2	0.152	730.4	67.1	0.041	325.6	78.2	0.0044	54.2

EAP = East Asia & Pacific, CA = Central Asia, HIC = High-income countries, LAC = Latin America & Caribbean, MENA = Middle East & North Africa, SA = South Asia, SSH-HIV = Sub-Saharan Africa High HIV, SSA = Other Sub-Saharan African countries. Source: Authors’ calculations based on UN World Population Prospects data.

Shifting our attention to adult mortality (see the second group of columns in Table 1), we find relatively similar trends. The average length of life above age 15 (*μ*_15_) tends to increase virtually in all places across all time periods but not as fast as life expectancy at birth increases. As in the previous case, the groups of high-income and Sub-Saharan African countries are the regions with the highest and lowest levels of *μ*_15_, respectively. Simultaneously, we observe generalized declines in adult lifespan variability—albeit at a much slower pace than the declines in overall lifespan inequality. There are some exceptions to this generally favorable trend in Central Asia, the high-income group around the 1990s (arguably as a consequence of the collapse of the Eastern bloc countries included in these regions), and the HIV-stricken Sub-Saharan African countries. For the last group, we observe some stagnation in the lifespan inequality declines around the 1990s and a slight inconsistency in the trends reported by the Theil index and the variance. The regional trends in overall and adult lifespan inequality reported in Table 1 are roughly consistent with the findings reported by Edwards [19] in his Fig 3.

Lastly, the trends in elderly mortality are notably different (see the third group of columns in Table 1). As expected, the average length of life above 65 (*μ*_65_) continues to increase in the world and most of its regions, but some regions increase faster than others. Interestingly, lifespan inequality among the elderly tends to *increase* over time for the world and all its regions (except in Central Asia): no matter which inequality indicator we use, we observe unequivocal increases in length of life variability in the older ages. Curiously, in contrast to the other lifespan inequality indicators shown in Table 1, the levels of lifespan inequality among the elderly across regions are relatively similar. In the 1950s, the group of high-income countries and Central Asia had the largest elder lifespan inequality, but in 2010, inequality was largest in Latin America and the Caribbean. These findings—which cohere with the results of Engelman, Canudas-Romo and Agree [5] in the context of high-income countries—uncover an extremely interesting pattern that, as we will now see, is also observed within the majority of world countries.

### Within countries lifespan inequality (WLI)

**Fig 2:**
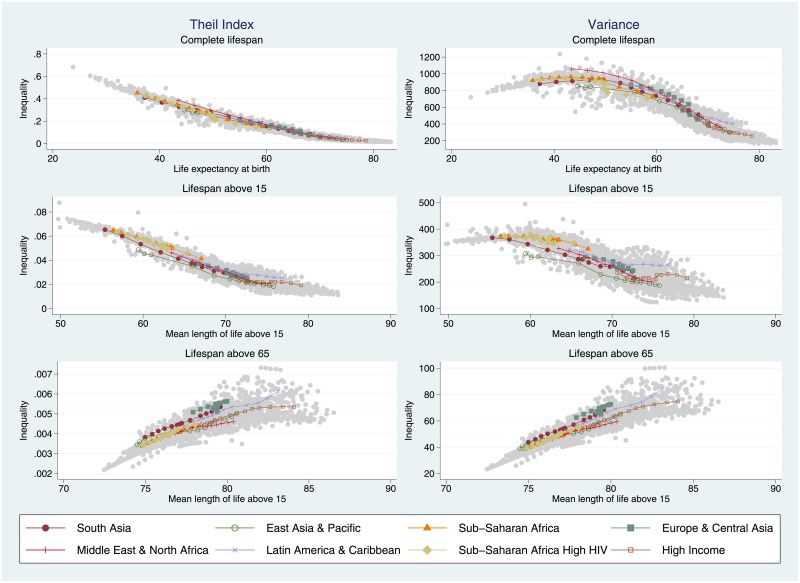
Scatterplots of longevity (horizontal axis) versus lifespan inequality (vertical axis) using the Theil index and the variance for overall, adult and elderly populations. Source: Authors’ calculations based on UN World Population Prospects data.

In our previous analysis, we explored the regional trends in longevity and lifespan inequality. What can be said about the experience of individual countries? In Fig 2, we show a 3×2 scatterplot matrix comparing longevity levels (horizontal axes) against the corresponding lifespan inequality indicators (vertical axes) using data from all world countries between 1950–55 and 2010–15. The scatterplots in the first, second and third rows are based on unconditional, above 15 and above 65 age-at-death distributions, respectively. The scatterplots on the first and second columns measure lifespan inequality using the Theil index and the variance, respectively. In all cases, we superimpose the regional trends for comparative purposes. In general, the trends shown in Fig 2 are in line with the trends presented in Table 1. As in previous studies such as Smits and Monden [4] and Edwards [19], we observe a strong negative correlation between life expectancy at birth and unconditional lifespan inequality (see first row in Fig 2). As the epidemiologic transition unfolds, longevity increases in tandem with decreases in lifespan inequality. Interestingly, all regions seem to follow a very similar path of demographic convergence, although we observe more cross-country heterogeneity when using absolute measures than relative ones.

Inspecting the relationship between longevity and *adult* mortality (i.e., disregarding under 15 mortality), a different picture arises (see second row in Fig 2). In this case, there is also a generally negative relationship between the two variables, but it is much weaker and the variability across countries and regions is substantially larger than before. Indeed, it is possible to identify several countries and regions where inequality declines stall and are followed by extended plateaus (this is the case for high-income countries as well as Latin America and the Caribbean). Once again, there is more between-country and between-region variability when using absolute inequality measures than there is when using relative measures. Lastly, examining the relationship between countries’ longevity and lifespan inequality among the elderly, we observe *diverging* trends across the board (see third row in Fig 2): as world countries’ longevity increases, the variability in age-at-death distributions among the elderly increases as well. The validity of this interesting result does *not* depend on the choice of inequality measure.

### Between-country and global lifespan inequalities (BLI and GLI)

**Fig 3:**
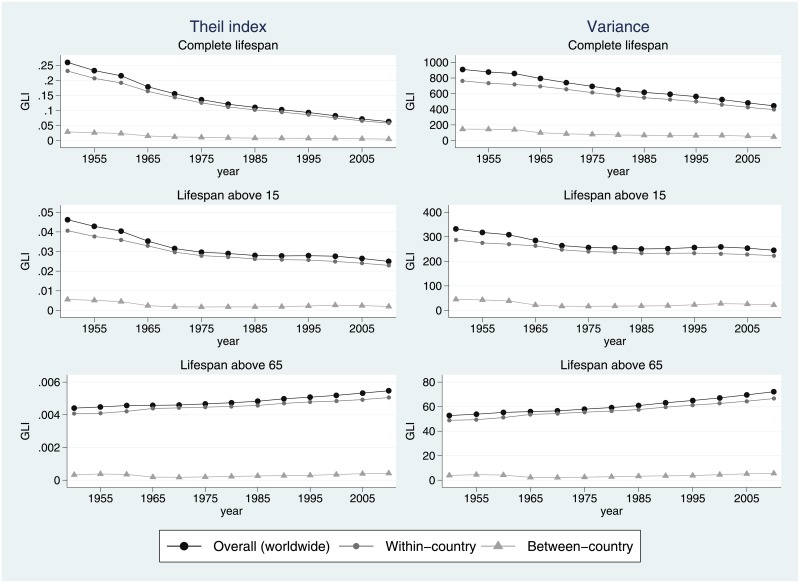
Global lifespan inequality between 1950–55 and 2010–15 using the Theil index and the variance (left and right columns, respectively) for overall, adult and elderly populations. Source: Authors’ calculations based on UN World Population Prospects data.

What can we say about the trends in global lifespan inequality? To what extent are these trends determined by length of life differences within and between countries? What are the contributions of the intra and intercountry disparities to GLI? Fig 3 plots the trends in GLI and its within- and between-country components between 1950–55 and 2010–15 (the values upon which this Figure is based are shown in S1 File). In the first row, we show the results corresponding to the entire age-at-death distributions, while the second and third rows show the results for the distributions bottom-truncated at 15 and 65, respectively. When considering unconditional age-at-death distributions, lifespan inequality has clearly declined over time—a result that does not depend on the choice of inequality measure. After six decades, GLI levels have shrunk dramatically from 0.26 to 0.06 for the Theil index and from 911.5 to 444.1 for the variance. Such concentration in the age-at-death distributions goes hand-in-hand with generalized increases in life expectancy at birth—a finding that squares well with related findings reported in previous studies [4, 6, 19]. Interestingly, most of the variation in lifespan across world citizens can be attributed to differences occurring *within* countries. The contribution of the between-country component for the Theil index goes from 11% in 1950–55 to 7.6% in 2010–15 (for the variance, it declines from 16% to 10.7%).

The values of GLI for the adult population are declining as well, but much less than in the previous case. The Theil index (resp. the variance) declines from 0.046 to 0.025 (resp. from 333.2 to 244.7). In both cases, we observe a clear decline between 1950–55 and 1980–85, followed by a long inequality plateau. In the case of the variance, we even observe some slight increases at the turn of the millennium, not only in some specific regions such as the Eastern European countries, the HIV-stricken countries of Sub-Saharan Africa, and countries in Latin America and the Caribbean but also in the world as a whole. These results suggest that the expected global compression in adult mortality has stagnated during the last 30–35 years or so. These results are in line with the findings of Edwards and Tuljapurkar [3] in reporting adult lifespan inequality plateaus for a selected group of highly industrialized countries. Most of these changes have taken place despite the generalized increases in longevity among the adult population. Again, the contribution of the between-country component is relatively minor (approximately 6–12% for the Theil and 6–13% for the variance). Between-country inequality in adult mortality decreased between 1950–55 and 1970–75, increased between 1970–75 and 2000–05 and declined again from 2000–05 until 2010–15. The description given in [19] for the period between 1970 and 2000 (identifying the rising between-country inequality in adult length-of-life) fits well with our findings, which provide a longer and more nuanced time perspective of the recent trends in international health inequality (during the last decade, between-country inequality in adult lifespan has resumed a *declining* trajectory).

Regarding the levels of GLI for age-at-death distributions above 65, we observe the opposite trend. During the last six decades, lifespan inequality among the world elderly has *increased* from 0.0044 to 0.0055 for the Theil index and from 52.6 to 72 for the variance (with Latin American and high-income countries exhibiting the highest levels of inequality). Once again, these global differences can be mainly attributed to the disparities occurring within countries. The within-country component of global lifespan inequality among the elderly has increased during the whole period, while the between-country component declined between 1950–55 and 1970–75 and started increasing unabated from 1970–75 until 2010–15—a 40-year divergence pattern across the countries.

### Sex-specific results

As done in conceptually related studies (e.g., Edwards [19]), the empirical results presented in this paper document lifespan inequality trends for the population as a whole (i.e., using both sexes’ life tables). However, it is well known that (a) women tend to survive to older ages than men and (b) age-at-death distributions tend to exhibit greater variability among men ([4, 14]). However, calculating our indicators for women and men separately, we observe that sex-specific trends in lifespan inequality follow the same general patterns we have identified in this study for the general population. For the sake of simplicity, we have preferred to avoid the duplicity of tables and graphs leading to the same qualitative conclusions (for the interested reader, the sex-specific results are available upon request).

### Counterfactual analysis

During the last six decades, the world has undergone major sociodemographic transformations. Both the population size and the rate of increase of longevity have varied considerably across countries. In addition, the shapes of the lifespan distributions have changed substantially over time. In this swiftly changing context, it is important to evaluate which of these explanatory factors have been more decisive in driving changes to GLI levels. For that purpose, we have run several counterfactual analyses. Using Eqs (2)–(5), we compare the real trends of GLI with the ones that would have been observed had some of its subcomponents (countries’ population shares, longevity and lifespan variability) remained constant over time. Table 2 reports such counterfactual trends, both for the Theil index and the variance and for conditional and unconditional age-at-death distributions.

**Table 2:** Counterfactual analyses for the Theil index and the variance.

**Theil counterfactuals**							
Year	1950–55	1960–65	1970–75	1980–85	1990–95	2000–05	2010–15
*Theil*	0.2629	0.2183	0.1571	0.1216	0.1028	0.0827	0.0629
*C*_1_ (*constant population*)		0.2153	0.1504	0.1126	0.0922	0.0713	0.0520
*C*_2_ (*constant μ*)		0.2126	0.1443	0.1071	0.0878	0.0684	0.0497
*C*_3_ (*constant T*_*w*_)		0.2664	0.2693	0.2782	0.2881	0.2970	0.3036
*Theil* (15+)	0.0466	0.0407	0.0316	0.029	0.0277	0.0275	0.0249
*C*_1_ (*constant population*)		0.0403	0.0307	0.0277	0.0263	0.0254	0.0225
*C*_2_ (*constant μ*)		0.0400	0.0305	0.0277	0.0264	0.0258	0.0227
*C*_3_ (*constant T*_*w*_)		0.0464	0.0454	0.0467	0.0479	0.0497	0.0500
*Theil* (65+)	0.0044	0.0046	0.0046	0.0047	0.005	0.0052	0.0055
*C*_1_ (*constant population*)		0.0046	0.0047	0.0049	0.0051	0.0053	0.0056
*C*_2_ (*constant μ*)		0.0046	0.0047	0.0049	0.0051	0.0052	0.0055
*C*_3_ (*constant T*_*w*_)		0.0044	0.0041	0.0041	0.0041	0.0042	0.0042
**Variance counterfactuals**							
*Variance*	911.5	860.8	744.9	650.3	593.0	525.5	444.1
*C*_1_ (*constant population*)		856.3	727.8	621.3	555.0	478.1	392.9
*C*_2_ (*constant μ*)		862.9	794.4	709.5	655.2	588.6	526.1
*C*_3_ (*constant V*_*w*_)		912.7	870.1	867.5	872.4	879.6	868.9
*Variance*(15+)	333.2	309.6	264.4	254.6	251.5	257.9	244.7
*C*_1_ (*constant population*)		307.7	260.5	248.1	245.1	246.4	231.2
*C*_2_ (*constant μ*)		314.6	289.5	278.6	273.7	272.2	265.2
*C*_3_ (*constant V*_*w*_)		330.1	312.6	317.4	322.9	335.2	333.3
*Variance*(65+)	52.6	55.2	56.5	59.1	63.0	67.0	72.0
*C*_1_ (*constant population*)		55.5	57.9	61.7	66.1	69.9	74.9
*C*_2_ (*constant μ*)		54.8	58.8	61.0	64.2	67.3	71.2
*C*_3_ (*constant V*_*w*_)		52.8	48.6	48.4	48.6	49.2	50.1

Source: Authors’ calculations based on UN World Population Prospects data.

Considering the entire age range, we can conclude that within-country inequality is the strongest determinant of the observed declines in GLI (see counterfactual*C*_3_). Had within-country inequality remained at its 1950–55 levels, GLI levels would have been much higher than the observed ones (i.e., from the “true” 0.0629 for the Theil in 2010–15 up to 0.3036 and from the “true” 444.1 for the variance in 2010–15 up to 868.9). At the other extreme, had population shares remained at their 1950–55 levels, global lifespan inequality would be slightly smaller than it is today (see counterfactual*C*_1_). Hence, even if population growth *per se* has contributed to widening the global lifespan distribution, its effect has been quantitatively small. Somewhere in between, we observe that the effect of longevity on GLI depends on the choice of inequality measure. For the Theil index, changes in longevity have slightly deterred further declines in GLI (i.e., fixing longevity at its 1950–55 levels, GLI would have reached 0.049 rather than the observed 0.06), while the opposite effect is found for the variance (see counterfactual*C*_2_). The counterfactual analyses applied to the age-at-death distributions bottom-truncated at the age of 15 are qualitatively very similar to the previous ones (see central rows in the two panels of Table 2). Lastly, the results for the distributions truncated at 65 suggest that neither population growth nor longevity changes have had an important effect in driving GLI trends. Once again, lifespan inequality trends within countries seem to be the major factor behind the observed GLI trends. Had within-country inequality levels remained fixed at their 1950–55 levels, GLI levels among the elderly would have barely changed during the last sixty years.

In summary, the empirical evidence presented here suggests that the *changes* in global lifespan inequality have been mainly driven by *changes* in within-country lifespan variability and, to a much lesser extent, by longevity trends across countries. Population growth has played a minor role in this process.

## Discussion and concluding remarks

In this paper, we document for the first time global trends in lifespan inequality from the 1950s to the present day. Our findings indicate that (i) there has been a sustained decline in overall lifespan inequality, (ii) adult lifespan variability has also declined, but some plateaus and trend reversals have been identified, and (iii) lifespan inequality among the elderly has *increased* virtually everywhere. All these changes have occurred against a backdrop of generalized mortality reductions. While such an increase in elderly lifespan inequality should be expected in the context of increasing longevity, it is nonetheless important to document which countries or regions are spearheading the process and which ones are lagging behind. The increase in lifespan variability among the elderly was previously investigated in a selected group of highly industrialized countries [5]. According to the authors of that study, the systematic increases in longevity alter the health profile of survivors in fundamental ways: advances in medicine, socioeconomic conditions and public health planning have facilitated frailer individuals reaching more advanced ages, thus increasing the heterogeneity in health profiles among the elderly. As shown in this paper, it turns out that such mechanisms might have been operating not only in high-income settings but also across all world countries and regions (irrespective of their stage in the demographic or epidemiological transitions).

Decomposing global lifespan inequality levels into within- and between-country components, we observe that most of the world variability in ages at death can be explained by differences occurring *within* countries. Depending on the inequality measure and the period we choose, the within-country component explains approximately 85% and 95% of the total variation (Smits and Monden [4] and Edwards [19] report analogous contributions within that range). This suggests that traditional narratives in global health disparities focusing on international variations in life expectancy (e.g., Moser, Shkolnikov and Leon [18], Goesling and Firebaugh [30]) neglect the major source of lifespan inequality: the source that takes place within countries. This is precisely the component that has experienced the most dramatic changes during the last six decades. Indeed, our counterfactual analyses suggest that the observed *changes* in global lifespan inequality can be largely attributable to the changes in within-country lifespan distributions, while the contributions of increasing longevity and differential population growth have played a relatively minor role. While the between-country component is relatively small, this does not mean it is irrelevant. The mere existence of a between-country component means that some individuals are expected to live longer than others simply because of their place of residence, a circumstance which they cannot be held accountable for.

### Sources of lifespan inequality

What factors might be driving these remarkable trends in lifespan inequality? Researchers have advanced several explanations for the determinants of international health inequalities (i.e., differences in longevity *between* countries). In an attempt to overcome the limitations of Omran’s epidemiological transition theory ([33–35] put forward the framework of ‘divergence-convergence cycles’. According to these authors, health transitions can be seen as a succession of cycles composed of divergence periods (generated by revolutionary health innovations, such as the eradication of infectious diseases, or the cardiovascular revolution), followed by the convergence that ensues when laggard countries adapt and catch up with the frontrunners. Indeed, the nonmonotonic trends observed in adult and elderly lifespan inequality between countries (see middle and bottom panels in Fig 3) fit well with that description. Very often, the diffusion of knowledge and the adoption of new technologies are listed among the key drivers of international health convergence. However, the evolution of such cycles can be suddenly interrupted when socioeconomic, political or other external shocks disrupt them for any reason. In this regard, the collapse of the Eastern Bloc and the spread of HIV/AIDS among Sub-Saharan African countries have been held responsible for the global increase in international health disparities around the 1990s [30]. Finally, socioeconomic differentials can be another key factor that might explain longevity variations across countries. In this line, the increasing cross-country disparities in elderly longevity might be partially explained by countries’ unequal access to increasingly expensive technologies that further prolong the lifespan of elderly populations.

**Fig 4:**
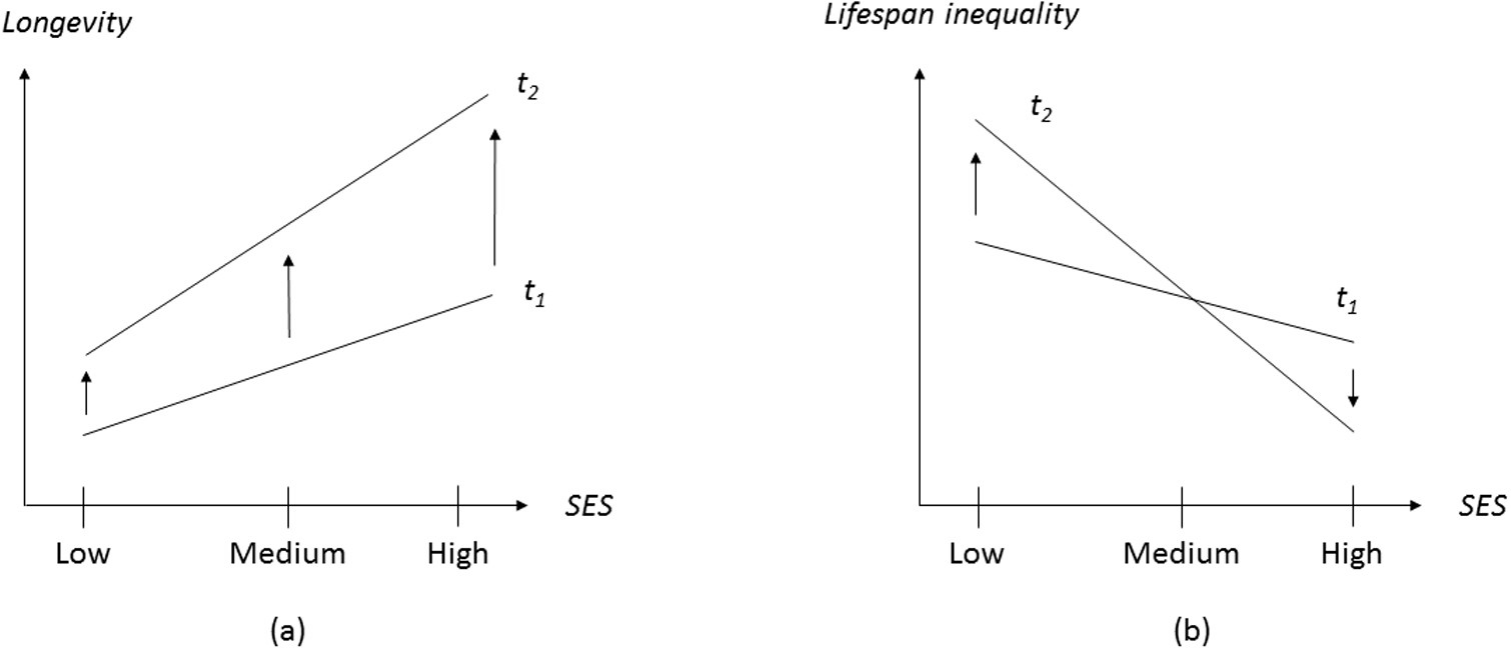
Within-country changes in longevity and lifespan inequality across SES groups over time.

As shown before, most global lifespan inequality changes have taken place *within* countries. The fundamental factor that has contributed to reducing countries’ lifespan variability is the reduction in infant mortality. This decrease has been extensively documented elsewhere [36–37] and can be largely attributed to the use of cheap and widely available treatments, such as the use of oral rehydration and antibiotics. Among adults and the elderly, within-country disparities in lifespan are often associated with the existence of socioeconomic gradients. The positive association between socioeconomic status (SES) and adult health and survival is well established [38–39]. To illustrate, higher-educated individuals are, through their higher income, more able to afford food, clothing and accommodation; have jobs that entail fewer health risks; are more engaged in healthy life styles and are better informed in using health services and new medical treatments [40–41]. In this regard, a collection of case studies in high-income settings suggests a clear patterning of longevity and lifespan variability within countries’ SES groups along the following lines. On the one hand, researchers have often found diverging longevity trends across SES groups, with the socially advantaged groups benefiting more than the rest ([42–47]; see left panel in Fig 4). On the other hand, a handful of studies have suggested that (i) there is a negative gradient between SES and lifespan inequality (i.e., lower socioeconomic groups tend to have higher levels of lifespan inequality) and (ii) the gradient becomes steeper over time because of the decrease (resp. increase) in lifespan variability among high (resp. low) SES groups (e [10–11, 48–49]; see right panel in Fig 4). Overall, these findings suggest the emergence of divergent health dynamics across SES groups within national borders (at least in the context of high-income countries). Whether these diverging patterns are also taking place in middle- and low-income countries is a matter for future research.

### Limitations and conclusions

The analysis presented in this paper has some limitations. First, all our findings are based on the worldwide life tables provided by the UN Population Division, some of which are based on estimated data. The indirect methods that are usually employed to estimate life tables based on incomplete information might oversmooth the corresponding age-at-death distributions—a potential source of downward bias for our lifespan inequality estimates. While we acknowledge that such bias might affect our estimates of lifespan inequality *levels* to a certain extent, we contend that it is less likely that the bias affects lifespan inequality *trends*, which are the main subject of interest in this paper. In addition, comparing lifespan inequality levels for those countries simultaneously included in the UN database *and* in the Human Mortality Database shows an extremely high level of correlation (S1 File). Second, the UN life tables are constructed up to age 100, while the HMD life tables include ages up to 110, an issue that might downwardly bias our lifespan inequality estimates. Once again, robustness checks presented in S1 File show that this source of bias is negligibly small. Lastly, our counterfactual lifespan inequality analyses might look somewhat crude at first sight. Using ceteris-paribus-like arguments, the analyses simply assume that some of the three components in our inequality measures can be kept fixed while the others are allowed to change over time as they actually did—in other words, as if they were completely independent entities. Reality is far more complex than that, and intricate relationship patterns bind the different components with one another. Despite this limitation, such techniques are very useful for deriving first-order approximations of complex phenomena that can otherwise only be approximated realistically with sophisticated models whose specifications depend on arbitrary decisions and that are themselves prone to a wide range of conceptual and measurement errors—factors that explain their popularity in demographic studies ([30, 50–51]).

Despite those limitations, the results presented in this paper confirm that the study of health inequalities should not be limited to the analysis of differences in life expectancy across countries. Since most lifespan variability takes place within countries, focusing on the trends of central longevity indicators alone disregards the major source of variability, thus potentially arriving at overly simplistic conclusions. During recent decades, much progress has been made in increasing longevity while reducing age-at-death variability across the full lifespan and, to a lesser extent, across adult ages. However, we now appear to face a new challenge: the emergence of diverging trends in lifespan inequality among the elderly *around the globe*. While lifespan inequality is increasing among the elderly across virtually all world countries, longevity and heterogeneity in mortality among the old has increased faster in the richer regions of the globe. As larger fractions of the world population survive to more advanced ages, it will be necessary for national and international health planners to recognize the growing heterogeneity that characterizes older populations.

## Supporting information

S1 File (DOCX)
