# Supplementary material for: Global trends in lifespan inequality: 1950-2015
Source: PLoS One. 2019 May 2;14(5):e0215742. doi: 10.1371/journal.pone.0215742 (PMC6497240; doi:10.1371/journal.pone.0215742)
Supplement: S1 File — (DOCX) [file pone.0215742.s001.docx]

**Supplementary Material**

*Countries’ regional classification (United Nations)*

**East Asia & Pacific:**

Brunei Darussalam, Cambodia, China, Fiji, French Polynesia, Guam, Indonesia, Kiribati, Korea (North), Korea (South), Lao PDR, Macao, SAR China, Malaysia, Micronesia, Federated States of, Mongolia, Myanmar, New Caledonia, Papua New Guinea, Philippines, Samoa, Singapore, Solomon Islands, Thailand, Timor-Leste, Tonga, Vanuatu, Viet Nam.

**Central Asia:**

Kazakhstan, Kyrgyzstan, Tajikistan, Turkmenistan, Uzbekistan.

**High Income:**

Albania, Australia, Austria, Belarus, Belgium, Bosnia and Herzegovina, Bulgaria, Canada, Croatia, Cyprus, Czech Republic, Denmark, Estonia, Finland, France, Germany, Greece, Hungary, Iceland, Ireland, Israel, Italy, Japan, Latvia, Lithuania, Luxembourg, Macedonia, Republic of, Moldova, Montenegro, Netherlands, New Zealand, Norway, Poland, Portugal, Romania, Russian Federation, Serbia, Slovakia, Slovenia, Spain, Sweden, Switzerland, Ukraine, United Kingdom, United States of America.

**Latin America & Caribbean**

Antigua and Barbuda, Argentina, Aruba, Bahamas, Barbados, Belize, Bolivia, Brazil, Chile, Colombia, Costa Rica, Cuba, Dominican Republic, Ecuador, El Salvador, French Guiana, Grenada, Guadeloupe, Guatemala, Guyana, Haiti, Honduras, Jamaica, Martinique, Mexico, Nicaragua, Panama, Paraguay, Peru, Puerto Rico, Saint Lucia, Saint Vincent and Grenadines, Suriname, Trinidad and Tobago, Uruguay, Venezuela (Bolivarian Republic), Virgin Islands.

**Middle East & North Africa**

Algeria, Armenia, Azerbaijan, Bahrain, Egypt, Georgia, Iraq, Jordan, Kuwait, Lebanon, Libya, Morocco, Oman, Palestinian Territory, Qatar, Saudi Arabia, Syrian Arab Republic (Syria), Tunisia, Turkey, United Arab Emirates, Yemen.

**South Asia:**

Afghanistan, Bangladesh, Bhutan, India, Iran, Islamic Republic of, Maldives, Nepal, Pakistan, Sri Lanka.

**Sub-Saharan Africa:**

Angola, Benin, Cape Verde, Chad, Comoros, Congo (Brazzaville), Congo, (Kinshasa), Cote d'Ivoire, Djibouti, Eritrea, Gambia, Guinea, Madagascar, Mali, Mauritania, Mauritius, Mayotte, Niger, Reunion, Sao Tome and Principe, Senegal, Seychelles, Sierra Leone, Somalia, United Republic of Tanzania.

**Sub-Saharan Africa High HIV:**

Botswana, Burkina Faso, Burundi, Cameroon, Central African Republic, Equatorial Guinea, Ethiopia, Gabon, Ghana, Guinea-Bissau, Kenya, Lesotho, Liberia, Malawi, Mozambique, Namibia, Nigeria, Rwanda, South Africa, South Sudan, Swaziland, Togo, Uganda, Zambia, Zimbabwe.

*Formulas for the Theil index and the Variance*

The Theil index can be written as:

$$T_{a}=\frac{1}{l_{a}}\sum_{x=a}^{\omega} d_{x}\left( \frac{\alpha_{x}}{\mu_{a}} \right)log\left( \frac{\alpha_{x}}{\mu_{a}} \right) (EQA1)$$

where $a$ and $\omega$ are the youngest and oldest age intervals taken from the life table, $l_{a}$ is the radix of the population, $\mu_{a}$ is the average age at death of the population, and $d_{x}$ and $\alpha_{x}$ are the life table number of deaths and the average age at death in the age interval $x$ to $x+5$, respectively.

While the Theil index has been mainly employed to measure economic inequality, its use is also widespread in information theory, physics, and, more recently, in demography. For instance, Smits and Monden (2009), Edwards (2011) and Permanyer et al (2018) have employed the Theil index in their study of lifespan inequality. While the interpretation of $T_{a}$ might not be very intuitive (according to Sen (1997), ‘[the Theil index] is not a measure that is exactly overflowing with intuitive sense’), it satisfies all the basic axioms one might expect from an inequality index (see Shorrocks (1980)) and, like all such measures, it simply summarizes the dispersion of a given distribution in a single number. Alongside other inequality measures, the Theil index has become widely used not because of its simple interpretation, but owing to its nice additive decomposability property, that allows breaking down total inequality in two clearly interpretable components: the within-group component and the between group component (see section #3 in the paper). As can be seen below (S2 table), the values of the Theil index are highly correlated with other popular inequality measures, like the Gini coefficient, or the coefficient of variation.

Using the same notation as before, the Variance can be written as:

$$V_{a}=\frac{1}{l_{a}}\sum_{x=a}^{\omega} d_{x}\left( \alpha_{x}-\mu_{a} \right)^{2} (EQA2)$$

*Computing the counterfactuals and additively decomposing the Theil index and the Variance.*

In order to compute the counterfactuals shown in equations (EQ3), (EQ4) and (EQ5), we need to write the Theil index and the variance in an appropriate form that explicitly shows the dependency of these measures on the following three factors: (i) population shares ($s_{c}$), (ii) longevity ($\mu_{c}$), and (iii) lifespan variability ($I_{c}$). In doing so, we show how these two measures can be written in its additively decomposable form (i.e. where total inequality is a neat sum of a within-group and a between-group component).

We start with the Theil index. Assuming we have a list of $n$ countries (indexed by$c$), it is well-known that the Theil index at time $t$ can be written as

$$T_{t}=\sum_{c=1}^{n} s_{c,t}\frac{\mu_{c,t}}{\mu_{t}}ln\left( \frac{\mu_{c,t}}{\mu_{t}} \right)+\sum_{c=1}^{n} s_{c,t}\frac{\mu_{c,t}}{\mu_{t}}T_{c,t} (EQA3)$$

The first part in equation (EQA3) is the between-country component (which is obtained assuming all individuals in each country die at the same age, so there is no within- country variation) and the second one is the within- country component (which is a weighted sum of the within-country inequalities). Observing that global average age at death is equal to the population-weighted sum of country-specific average age at deaths,$\mu_{t}=\sum_{c=1}^{n} s_{c,t}\mu_{c,t}$ the additive decomposition of the Theil index (shown in (EQA1)) for time ‘$t$’ can be rewritten as:

$$T_{t}=\sum_{c=1}^{n} s_{c,t}\frac{\mu_{c,t}}{\sum_{c=1}^{n} s_{c,t}\mu_{c,t}}ln\left( \frac{\mu_{c,t}}{\sum_{c=1}^{n} s_{c,t}\mu_{c,t}} \right)+\sum_{c=1}^{n} s_{c,t}\frac{\mu_{c,t}}{\sum_{c=1}^{n} s_{c,t}\mu_{c,t}}T_{c,t} (EQA4)$$

In equation (EQA4) we explicitly see how the Theil index in time ‘$t$’ can be written as a function of countries’ population shares ($s_{c,t}$), longevity ($\mu_{c,t}$), and lifespan variability ($T_{c,t}$).

As regards the variance, it is also well-known that it can be written as

$$V_{t}=\sum_{c=1}^{n} s_{c,t}\left( \mu_{c,t}-\mu_{t} \right)^{2}+\sum_{c=1}^{n} s_{c,t}V_{c,t} (EQA5)$$

This is the additive decomposition of the variance. The first part in equation (EQA5) is the between-country component and the second one is the within- country component (which is a weighted sum of the within-country inequalities). After simple algebraic manipulations, the last equation can be written as

$$V_{t}=\sum_{c=1}^{n} s_{c,t}\mu_{c,t}^{2}-\mu_{t}^{2}+\sum_{c=1}^{n} s_{c,t}V_{c,t} (EQA6)$$

Once again, since $\mu_{t}=\sum_{c=1}^{n} s_{c,t}\mu_{c,t}$, we can finally rewrite the variance in time$t$ as

$$V_{t}=\sum_{c=1}^{n} s_{c,t}\mu_{c,t}^{2}-\left( \sum_{c=1}^{n} s_{c,t}\mu_{c,t} \right)^{2}+\sum_{c=1}^{n} s_{c,t}V_{c,t} (EQA7)$$

This way, we have written the global variance as a function of the three ingredients we were looking for: the vector of country-specific population shares ($s_{c,t}$), variances ($V_{c,t}$) and longevity levels ($\mu_{c,t}$).

*Decomposition of Global Lifespan Inequality*

The following table shows the levels and trends of global lifespan inequality and its decomposition in between-country and within-country inequality (Figure 3 is based upon these numbers).

**S1 Table. Theil and variance decompositions of Global Lifespan Inequality over time for the full age-at-death distribution, the deaths above 15 and above 65.**

**Theil decomposition of GLI**

| Year | 1950-55 | 1960-65 | 1970-75 | 1980-85 | 1990-95 | 2000-05 | 2010-15 |
| --- | --- | --- | --- | --- | --- | --- | --- |
| $Theil$ | 0.2629 | 0.2183 | 0.1571 | 0.1216 | 0.1028 | 0.0827 | 0.0629 |
| ${Theil}_{w}$ | 0.2341 | 0.1947 | 0.1449 | 0.1126 | 0.0950 | 0.0756 | 0.0581 |
| ${Theil}_{b}$ | 0.0288 | 0.0236 | 0.0122 | 0.0090 | 0.0078 | 0.0072 | 0.0048 |
| $\%Contr (btw.)$ | 10.9 | 10.8 | 7.7 | 7.4 | 7.6 | 8.6 | 7.6 |
| $Theil (15+)$ | 0.0466 | 0.0407 | 0.0316 | 0.0290 | 0.0277 | 0.0275 | 0.0249 |
| ${Theil}_{w}$ | 0.0410 | 0.0362 | 0.0298 | 0.0273 | 0.0259 | 0.0249 | 0.0229 |
| ${Theil}_{b}$ | 0.0056 | 0.0045 | 0.0018 | 0.0018 | 0.0019 | 0.0026 | 0.0020 |
| $\%Contr (btw.)$ | 12.0 | 11.0 | 5.7 | 6.1 | 6.7 | 9.5 | 8.0 |
| $Theil (65+)$ | 0.0044 | 0.0046 | 0.0046 | 0.0047 | 0.0050 | 0.0052 | 0.0055 |
| ${Theil}_{w}$ | 0.0041 | 0.0042 | 0.0044 | 0.0045 | 0.0047 | 0.0048 | 0.0051 |
| ${Theil}_{b}$ | 0.0003 | 0.0004 | 0.0002 | 0.0002 | 0.0003 | 0.0003 | 0.0004 |
| $\%Contr (btw.)$ | 7.6 | 7.8 | 3.8 | 4.7 | 5.5 | 6.6 | 7.6 |

**Variance decomposition of GLI**

| Year | 1950-55 | 1960-65 | 1970-75 | | 1980-85 | 1990-95 | | 2000-05 | 2010-15 |
| --- | --- | --- | --- | --- | --- | --- | --- | --- | --- |
| $Variance$ | 911.5 | 860.8 | 744.9 | | 650.3 | 593 | | 525.5 | 444.1 |
| ${Var}_{w}$ | 766.6 | 722.4 | 659.6 | | 578.9 | 528 | | 461 | 396.6 |
| ${Var}_{b}$ | 144.9 | 138.4 | 85.3 | | 71.4 | 65.0 | | 64.5 | 47.5 |
| $\%Contr (btw.)$ | 15.9 | 16.1 | 11.5 | | 11.0 | 11.0 | | 12.3 | 10.7 |
| $Variance (15+)$ | 333.2 | 309.6 | 264.4 | | 254.6 | 251.5 | | 257.9 | 244.7 |
| ${Var}_{w}$ | 288.4 | 271.1 | 247.4 | | 237.2 | 232.7 | | 230.7 | 222.9 |
| ${Var}_{b}$ | 44.8 | 38.5 | 17.0 | | 17.4 | 18.8 | | 27.2 | 21.8 |
| $\%Contr (btw.)$ | 13.4 | 12.4 | 6.4 | | 6.8 | 7.5 | | 10.5 | 8.9 |
| $Variance (65+)$ | 52.6 | 55.2 | 56.5 | 59.1 | | 63.0 | 67.0 | | 72.0 |
| ${Var}_{w}$ | 48.7 | 51.0 | 54.3 | 56.3 | | 59.5 | 62.6 | | 66.5 |
| ${Var}_{b}$ | 4.0 | 4.3 | 2.2 | 2.8 | | 3.5 | 4.4 | | 5.5 |
| $\%Contr (btw.)$ | 7.5 | 7.7 | 3.9 | 4.8 | | 5.5 | 6.6 | | 7.6 |

Source: Authors’ elaboration based on UN WPP data.

**Robustness checks**

To check the robustness of our empirical findings, we have performed different consistency tests. First, we have recalculated all our findings using well-known inequality measures other than the Theil index and the variance. Second, we have investigated whether or not the fact of working with life tables up to age 100 (rather than the value of 110 available in the HMD life tables) can downwardly bias our results.

*1. Use of alternative inequality measures*

Are our findings robust to the choice of alternative inequality measures? To check the robustness of our empirical findings, we will use other well-known inequality measures. One of them will be the family of Generalized Entropy measures GE(*θ*), which includes the Theil index as a particular case when $\theta=1$. It is defined as

$$\mathrm{GE}\left( \theta\right)=\left\{ \begin{matrix} \frac{1}{l_{a}}\frac{1}{\theta\left( \theta-1 \right)}\sum_{x=a}^{\omega} d_{x}\left[ \left( \frac{\alpha_{x}}{\mu_{a}} \right)^{\theta}-1 \right] if \theta\neq0,1 \\ \frac{1}{l_{a}}\sum_{x=a}^{\omega} d_{x}log\left( \frac{\mu_{a}}{\alpha_{x}} \right) if \theta=0 \\ \frac{1}{l_{a}}\sum_{x=a}^{\omega} d_{x}\left( \frac{\alpha_{x}}{\mu_{a}} \right)log\left( \frac{\alpha_{x}}{\mu_{a}} \right) if \theta=1 \end{matrix} (EQA8) \right.$$

The choice of different values of $\theta$ give more emphasis to different parts of the distribution^[[1]](#footnote-1)^. In S2 Table, we show the regional trends in GE(*θ*) when *θ=0* and *θ=2*. In addition, S2 Table shows the regional trends in lifespan inequality when using the Gini index and the coefficient of variation, which are defined as follows:

$$G=\frac{1}{2l_{a}^{2}\mu_{a}}\sum_{x=a}^{\omega} \sum_{x=a}^{\omega} d_{x}d_{y}\left| \alpha_{x}-\alpha_{y} \right| (EQA9)$$

$$CV=\frac{\sqrt{V_{a}}}{\mu_{a}} (EQA10)$$

|  |  | Full lifespan | | | | Ages 15+ | | | | Ages 65+ | | | |
| --- | --- | --- | --- | --- | --- | --- | --- | --- | --- | --- | --- | --- | --- |
| Region | Year | GE(0) | GE(2) | Gini | CV | GE(0) | GE(2) | Gini | CV | GE(0) | GE(2) | Gini | CV |
| EAP | 1950-55 | 0.7969 | 0.2154 | 0.3691 | 0.6564 | 0.0579 | 0.0437 | 0.1652 | 0.2955 | 0.0035 | 0.0036 | 0.0462 | 0.0850 |
|  | 1970-75 | 0.4546 | 0.0926 | 0.2221 | 0.4303 | 0.0324 | 0.0241 | 0.1183 | 0.2195 | 0.0041 | 0.0041 | 0.0507 | 0.0908 |
|  | 1990-95 | 0.2710 | 0.0507 | 0.1555 | 0.3185 | 0.0252 | 0.0187 | 0.1025 | 0.1934 | 0.0045 | 0.0045 | 0.0535 | 0.0952 |
|  | 2010-15 | 0.1199 | 0.0275 | 0.1152 | 0.2344 | 0.0213 | 0.0162 | 0.0955 | 0.1802 | 0.0050 | 0.0050 | 0.0562 | 0.0999 |
| ECA | 1950-55 | 0.4900 | 0.0987 | 0.2310 | 0.4442 | 0.0357 | 0.0262 | 0.1233 | 0.2289 | 0.0044 | 0.0044 | 0.0527 | 0.0940 |
|  | 1970-75 | 0.2197 | 0.0461 | 0.1526 | 0.3036 | 0.0294 | 0.0221 | 0.1132 | 0.2104 | 0.0046 | 0.0047 | 0.0542 | 0.0965 |
|  | 1990-95 | 0.1726 | 0.0414 | 0.1483 | 0.2879 | 0.0317 | 0.0242 | 0.1199 | 0.2200 | 0.0049 | 0.0049 | 0.0560 | 0.0992 |
|  | 2010-15 | 0.0972 | 0.0297 | 0.1269 | 0.2436 | 0.0285 | 0.0220 | 0.1137 | 0.2095 | 0.0053 | 0.0052 | 0.0578 | 0.1021 |
| HIC | 1950-55 | 0.3558 | 0.0718 | 0.1937 | 0.3790 | 0.0336 | 0.0248 | 0.1202 | 0.2228 | 0.0045 | 0.0046 | 0.0537 | 0.0955 |
|  | 1970-75 | 0.1981 | 0.0426 | 0.1458 | 0.2918 | 0.0276 | 0.0207 | 0.1093 | 0.2036 | 0.0049 | 0.0049 | 0.0558 | 0.0990 |
|  | 1990-95 | 0.1071 | 0.0282 | 0.1191 | 0.2373 | 0.0244 | 0.0184 | 0.1021 | 0.1916 | 0.0053 | 0.0053 | 0.0580 | 0.1025 |
|  | 2010-15 | 0.0635 | 0.0200 | 0.1005 | 0.1999 | 0.0201 | 0.0152 | 0.0921 | 0.1746 | 0.0054 | 0.0052 | 0.0578 | 0.1025 |
| LAC | 1950-55 | 0.7201 | 0.1691 | 0.3210 | 0.5815 | 0.0520 | 0.0385 | 0.1534 | 0.2773 | 0.0043 | 0.0043 | 0.0519 | 0.0931 |
|  | 1970-75 | 0.4844 | 0.0994 | 0.2333 | 0.4458 | 0.0372 | 0.0275 | 0.1272 | 0.2346 | 0.0046 | 0.0046 | 0.0540 | 0.0962 |
|  | 1990-95 | 0.2633 | 0.0559 | 0.1711 | 0.3343 | 0.0353 | 0.0261 | 0.1233 | 0.2283 | 0.0051 | 0.0052 | 0.0572 | 0.1017 |
|  | 2010-15 | 0.1492 | 0.0375 | 0.1398 | 0.2739 | 0.0323 | 0.0236 | 0.1165 | 0.2173 | 0.0061 | 0.0061 | 0.0625 | 0.1104 |
| MENA | 1950-55 | 1.1240 | 0.3043 | 0.4370 | 0.7802 | 0.0564 | 0.0415 | 0.1597 | 0.2880 | 0.0039 | 0.0039 | 0.0494 | 0.0888 |
|  | 1970-75 | 0.7129 | 0.1570 | 0.3039 | 0.5604 | 0.0438 | 0.0318 | 0.1371 | 0.2521 | 0.0041 | 0.0042 | 0.0510 | 0.0911 |
|  | 1990-95 | 0.3063 | 0.0600 | 0.1730 | 0.3464 | 0.0305 | 0.0222 | 0.1121 | 0.2109 | 0.0043 | 0.0044 | 0.0524 | 0.0933 |
|  | 2010-15 | 0.1647 | 0.0341 | 0.1270 | 0.2613 | 0.0240 | 0.0177 | 0.0987 | 0.1879 | 0.0045 | 0.0045 | 0.0532 | 0.0944 |
| SA | 1950-55 | 1.0375 | 0.3185 | 0.4536 | 0.7982 | 0.0768 | 0.0607 | 0.1990 | 0.3485 | 0.0038 | 0.0039 | 0.0484 | 0.0884 |
|  | 1970-75 | 0.7927 | 0.1913 | 0.3427 | 0.6185 | 0.0498 | 0.0369 | 0.1499 | 0.2715 | 0.0043 | 0.0045 | 0.0524 | 0.0943 |
|  | 1990-95 | 0.4986 | 0.1051 | 0.2411 | 0.4585 | 0.0380 | 0.0278 | 0.1278 | 0.2359 | 0.0047 | 0.0048 | 0.0545 | 0.0975 |
|  | 2010-15 | 0.2819 | 0.0567 | 0.1703 | 0.3367 | 0.0312 | 0.0233 | 0.1165 | 0.2160 | 0.0054 | 0.0055 | 0.0588 | 0.1047 |
| SSH-HIV | 1950-55 | 1.0868 | 0.3611 | 0.4796 | 0.8498 | 0.0765 | 0.0588 | 0.1948 | 0.3430 | 0.0033 | 0.0034 | 0.0455 | 0.0828 |
|  | 1970-75 | 0.8855 | 0.2586 | 0.4067 | 0.7192 | 0.0680 | 0.0506 | 0.1784 | 0.3180 | 0.0038 | 0.0039 | 0.0489 | 0.0882 |
|  | 1990-95 | 0.7151 | 0.1900 | 0.3448 | 0.6165 | 0.0614 | 0.0450 | 0.1668 | 0.3001 | 0.0041 | 0.0041 | 0.0508 | 0.0909 |
|  | 2010-15 | 0.4383 | 0.1045 | 0.2455 | 0.4571 | 0.0502 | 0.0362 | 0.1471 | 0.2692 | 0.0044 | 0.0044 | 0.0527 | 0.0938 |
| SSH | 1950-55 | 1.0505 | 0.3336 | 0.4621 | 0.8168 | 0.0754 | 0.0575 | 0.1922 | 0.3392 | 0.0034 | 0.0035 | 0.0458 | 0.0834 |
|  | 1970-75 | 0.7973 | 0.2172 | 0.3707 | 0.6591 | 0.0639 | 0.0470 | 0.1710 | 0.3067 | 0.0039 | 0.0039 | 0.0492 | 0.0887 |
|  | 1990-95 | 0.6779 | 0.1781 | 0.3343 | 0.5968 | 0.0622 | 0.0466 | 0.1708 | 0.3052 | 0.0041 | 0.0042 | 0.0509 | 0.0913 |
|  | 2010-15 | 0.4174 | 0.1050 | 0.2497 | 0.4583 | 0.0552 | 0.0408 | 0.1584 | 0.2857 | 0.0044 | 0.0045 | 0.0531 | 0.0946 |

**S2 Table. Lifespan inequality using different indicators across world regions between 1950-55 and 2010-15.** Source: Authors’ elaboration based on UN WPP data.

The results shown in S2 Table indicate that our findings are highly robust when using other inequality indices. Even if the inequality levels inevitably change when using alternative measures, the differences across regions and over time are preserved. No matter what inequality index we use, we observe generalized declines in age-at-death inequality for the complete lifespan, weaker declines (and even some trend reversals) when focusing on adult mortality, and generalized increases among the elderly.

*2. Upper limit of the life table*

To assess the robustness of our findings to the use of abridged life tables at age 100, we revisit our analysis with data from the Human Mortality Database (HMD) which contains life table up to age 110 for a limited numbers of (mostly high-income) countries. We find that, considering the full population of ages 0 to 110, the impact of lumping ages 100-110 together in a single category is very small: it amounts to less than 0.01 percent on average for all measures, with the smallest changes occurring for the Gini coefficients (which, as opposed to the other measures employed in the analysis, is not sensitive to the top-, but rather to the middle of the distribution). The largest percentage change observed across all measures, countries, and years is still only 0.5 percent and occurs for variance of the country with the highest life expectancy in the entire dataset, Japan, in the 2010-2015 time period. Naturally, if the population is restricted to older subpopulations, the impact of abridgment is larger, but still negligibly small: for the 65+ population, the changes on our inequality measures are all lower than .01 percent on average. Given that the countries in the HMD are among those with the highest life expectancies worldwide, the effects of abridging life tables at age 100 can be expected to be even smaller for less developed countries where only a very small part of the population survives above age 100 even in the more recent years. We therefore conclude that the use of abridged life tables has virtually no impact, neither qualitatively nor quantitatively, on the results obtained in this paper.

**References**

Edwards R. Changes in world inequality in length of life: 1970-2000. Popul Dev Rev*.*2011; 37(3): 499-528.

Permanyer I, Spijker J, Blanes A, Rentería E. Longevity and lifespan variation by educational attainment in Spain: 1960-2015. Demography. 2018; 55(6): 2045-2070.

Sen A. On Economic Inequality. Oxford: Clarendon Press. 1997.

Shorrocks A F. The Class of Additively Decomposable Inequality Measures. Econometrica. 1980; 48: 613-625.

Smits J, Monden C. Length of life inequality around the globe. Soc Sci Med. 2009; 68:1114-1123.

1. Lower values of $\theta$ are associated with greater sensitivity to inequality at the lower tail of the distribution (i.e. among children and young individuals), and higher values of $\theta$ place more weight to inequality among the elderly. When $\theta=0$ we obtain the so-called ‘mean log deviation’ (MLD) and when$\theta=2$, $\mathrm{GE}\left( 2 \right)$ is ordinally equivalent to the squared coefficient of variation. [↑](#footnote-ref-1)
